# Case Report: Abscopal Effect of Microwave Ablation in a Patient With Advanced Squamous NSCLC and Resistance to Immunotherapy

**DOI:** 10.3389/fimmu.2021.696749

**Published:** 2021-08-03

**Authors:** Chuchu Shao, Menghang Yang, Yingying Pan, Dacheng Xie, Bin Chen, Shengxiang Ren, Caicun Zhou

**Affiliations:** Department of Medical Oncology, Shanghai Pulmonary Hospital, Thoracic Cancer Institute, Tongji University School of Medicine, Shanghai, China

**Keywords:** ablation, immunotherapy, lung cancer, abscopal effect, oligo-progression

## Abstract

Currently, immunotherapy has been a backbone in the treatment of advanced non-small cell lung cancer (NSCLC) without driver gene mutations. However, only a small proportion of NSCLC patients respond to immune checkpoint inhibitors, and majority of patients with initial response will develop acquired resistance at 5 years, which usually manifests as oligo-progression or oligo-metastases. Evidence from multiple clinical trials indicates that local consolidative therapies could improve the prognosis of oligometastatic NSCLC patients. Herein, we reported a case of advanced squamous lung cancer which showed a durable abscopal effect from microwave ablation after acquired resistance of immunotherapy.

## Introduction

Lung cancer remains a malignant disease with high incidence and mortality (1). With the advent of immune checkpoint inhibitors, programmed cell-death protein 1 (PD-1) or programmed cell death 1 ligand 1 (PD-L1) blockade has become a backbone as first-line treatment, and a standard of care as second-line therapy for patients with advanced NSCLC and EGFR/ALK wildtype (2). However, the clinical outcomes of immunotherapies are not always satisfying. Schoenfeld AJ et al. found that 74% of patients with initial response to immunotherapy will develop acquired resistance at 5 years, and 56% of them experienced oligo-progression (3). In recent years, locoregional therapies have been widely used to treat patients with oligo-progression or oligo-metastases and showed impressive outcomes in multiple solid tumors (4, 5). Among these local therapies, microwave ablation (MWA) is increasingly used in clinical practice due to its advantages of producing larger ablation zones over shorter periods of time. Herein, we report an abscopal effect of MWA in a 69-year-old patient with metastatic squamous lung cancer. We performed a right lower lung lesion ablation after resistance of immunotherapy and observed tumor shrinkage in 4R/7 lymph node metastatic lesions. We posit that the present case report will provide novel insight into the treatment of advanced NSCLC in clinical practice.

## Case Presentation

**Figure 1:**
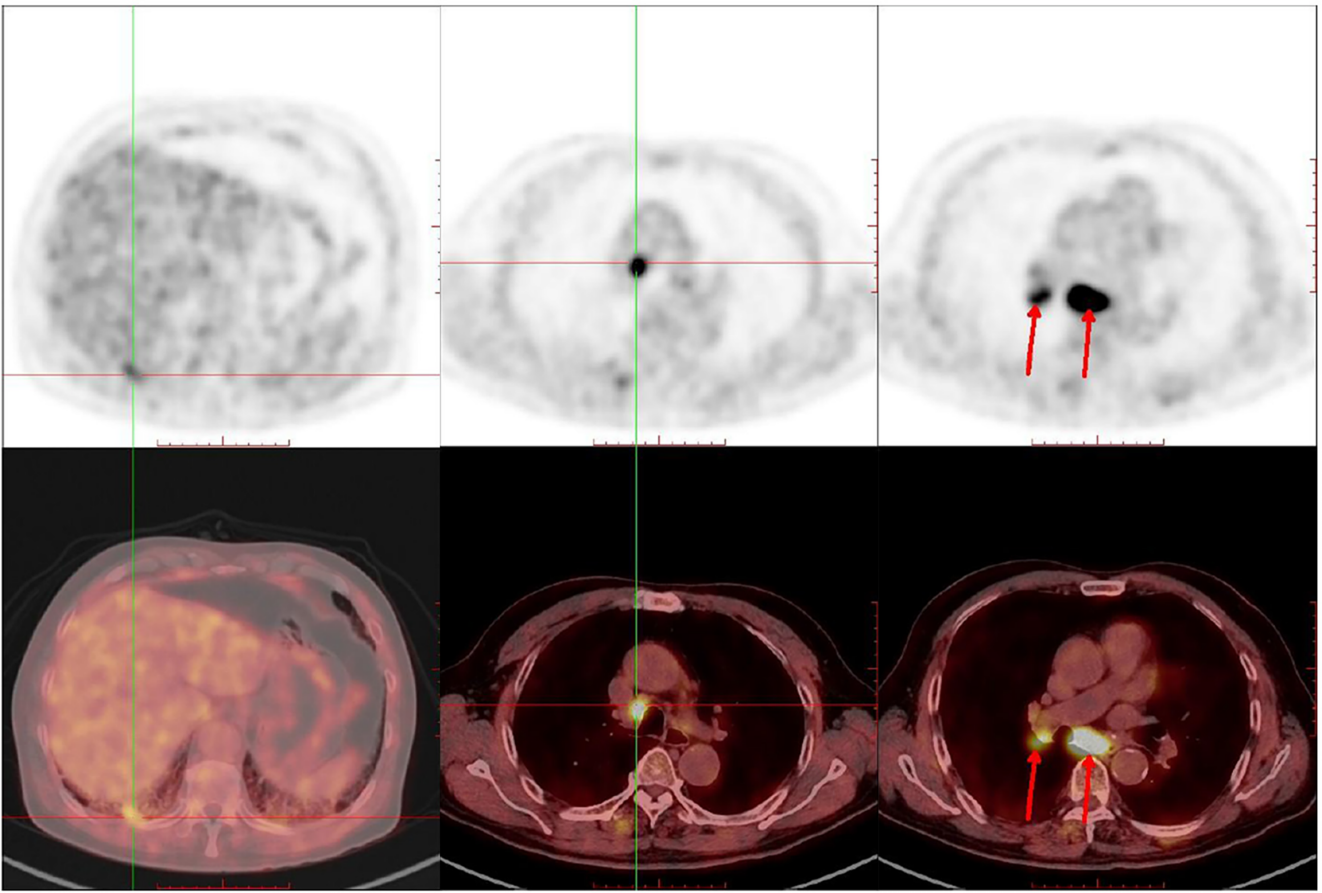
PET/CT revealed a right lower lobe mass and 4R/7 lymphadenopathy before treatment.

**Figure 2:**
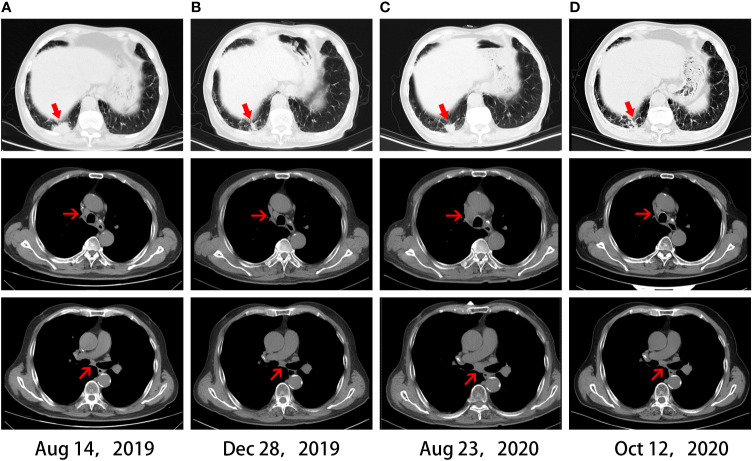
Chest CT scans. **(A)** CT before immunotherapy. **(B)** CT revealed a partial response after four months of immunotherapy. **(C)** CT revealed disease progression after acquired resistance of immunotherapy. **(D)** CT revealed an abscopal effect after one month of local ablation.

In June 2018, a 69-year-old male with a 30 pack–year smoking history was referred to Shanghai Pulmonary Hospital for a right lower lobe mass and 4R/7 lymphadenopathy (**Figure 1**), along with severe chronic obstructive pulmonary disease (COPD). Ultrasound-guided bronchial biopsy revealed squamous cell carcinoma, and genetic testing showed negativity for driver gene mutations. The patient was initially treated with vinorelbine 40 mg/m^2^ d1, 8 and cisplatin 60 mg/m^2^ d1–2. However, after four cycles, his symptoms worsened, and chest computed tomography (CT) scan confirmed a progressive disease (PD). Hence, the chemotherapy regimen was shifted to albumin-bound paclitaxel 200 mg/m^2^ d1, 8. The maximum diameter of primary lesion shrank 28% during the treatment of albumin-bound paclitaxel. However, after four cycles the primary lung lesion was still not effectively controlled, and disease progression in the chest was confirmed by imaging (**Figure 2A**), and ECT bone scan revealed a new lesion in the right tibia, indicating the occurrence of bone metastases. In addition, the PD-L1 expression of this patient showed negative results.

**Figure 3:**
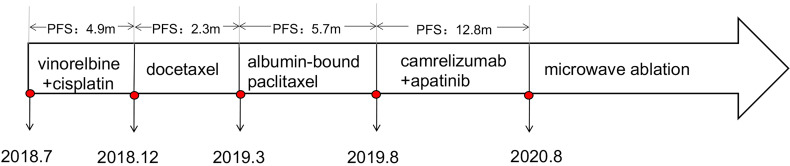
Timeline of treatment administration from the episode of care.

In August 2019, the patient participated in a single-arm phase II clinical study of camrelizumab plus apatinib for advanced NSCLC. After four cycles, a partial response (PR) was observed in December 2019 (**Figure 2B**), with a PFS of 12.8 months. Oligo-progression in the chest was found in August 2020, with enlarged primary lung lesion and mediastinal 4R/7 lymphadenopathy (**Figure 2C**). Given that this patient had severe COPD and could not tolerate radiotherapy in the lung and mediastinum simultaneously, CT-guided microwave ablation was utilized to eliminate the primary tumor in September 2020. One month later, chest CT scan showed the right lower lobe mass was gradually absorbed. Surprisingly, the enlarged 4R/7 lymph nodes shrank significantly at the same time and continued to decrease by subsequent follow-up scans, indicating an abscopal effect of local ablation (**Figure 2D**). Thus, we cancelled the original plan of radiotherapy for him and decided to continue anti-PD-1 immunotherapy as before. Until the last follow-up in March 2021, the patient had not shown any signs of disease progression and obtained a durable response. The timeline treatment administration from the episode of care was presented in **Figure 3**.

## Discussion

In this report, we presented a successful case of a patient with advanced squamous cell lung cancer who showed an abscopal effect of local ablation. This patient showed initial response to PD-1 blockade and VEGFR-TKI after the failure of traditional chemotherapy. However, he developed acquired resistance thereafter with an oligo-progression. Emerging evidence demonstrated that locoregional therapies improved overall survival in oligo-progressive NSCLC patients and became a standard therapeutic strategy after resistance to molecular targeted therapy (6, 7). In the present case, we applied local ablative therapy in oligo-progressive NSCLC after immunotherapy resistance and observed a durable abscopal effect, which highlighted the importance of local ablation in cancer immunotherapy.

Although thermal ablative techniques are becoming more frequent in lung cancer, the mechanism of their systemic immunomodulatory effects remains controversial. Thermal ablation mainly includes radiofrequency ablation (RFA), microwave ablation (MWA), and argon–helium knife cryotherapy (8). Among them, MWA showed favored features of shorter ablation times and potentially larger ablation zones (9, 10). Typically, MWA provides high thermal energy to cause tumor necrosis as an *in situ* antigen and thereby initiating systemic immune response, which is similar to radiation-induced abscopal effect (11). In addition, thermal ablation could change tumor microenvironment by promoting the infiltration of tumor-specific T cells. Previously, Zerbini et al. demonstrated that circulating tumor-specific T cells and natural killer (NK) cells were activated and enhanced by RFA that was applied to hepatocellular carcinoma (12). The increase of T cells, NK cells, or macrophages within the tumor microenvironment after thermal ablation needs to be validated by more experimental studies in the future.

To the best of our knowledge, this is the first case that showed a durable abscopal effect of MWA in squamous NSCLC after acquired resistance of immunotherapy. Local ablation eliminated the primary lesion and exerted an abscopal effect on the distant lesions by boosting the immune system; local ablation might provide a novel strategy for patients who developed acquired resistance to immunotherapy. NSCLC patients with multiple metastases might also benefit from local ablation therapy due to the appearance of abscopal effect. Therefore, the application of local ablative therapies showed a superior potency in the area of immunotherapy than targeted therapy. Additionally, the abscopal effects of radiotherapy have been observed in previous reports (13, 14), and local ablative therapies might be an alternative surrogate.

Nevertheless, there exist several limitations in our report. The application of MWA is still not widely used in clinical practice for lung cancer. Therefore, clinical trials that compare the efficacy of local ablation with other therapies, such as radiotherapy, are urgently needed. In addition, the mechanism of how local ablation stimulates abscopal effect after resistance to immunotherapy needs to be clarified further. Our case sheds light foroptimal approach in patients with lung cancer who developed acquired resistance to immunotherapy.

## Data Availability Statement

The original contributions presented in the study are included in the article/supplementary material. Further inquiries can be directed to the corresponding author.

## Ethics Statement

Written informed consent was obtained from the individual(s) for the publication of any potentially identifiable images or data included in this article.

## Author Contributions

CS and MY drafted the manuscript. YP, DX, and BC collected materials and prepared figures. SR and CZ critically revised the final manuscript. All authors contributed to the article and approved the submitted version.

## Funding

This research was supported by grants from the National Natural Science Foundation of China (No. 81772467, No. 81972167), Shanghai Pujiang Program (No. 2019PJD048), Shanghai Shenkang Hospital Development Center (No. SHDC12019133), Clinical Research foundation of ShangHai Pulmonary Hospital (No. FKLY20008).

## Conflict of Interest

The authors declare that the research was conducted in the absence of any commercial or financial relationships that could be construed as a potential conflict of interest.

## Publisher’s Note

All claims expressed in this article are solely those of the authors and do not necessarily represent those of their affiliated organizations, or those of the publisher, the editors and the reviewers. Any product that may be evaluated in this article, or claim that may be made by its manufacturer, is not guaranteed or endorsed by the publisher.
